# Photodynamic Antimicrobial Chemotherapy: Advancements in Porphyrin-Based Photosensitize Development

**DOI:** 10.3389/fchem.2021.635344

**Published:** 2021-04-07

**Authors:** James Oyim, Calvin A. Omolo, Edith K. Amuhaya

**Affiliations:** ^1^ School of Pharmacy and Health Sciences, United States International University-Africa, Nairobi, Kenya; ^2^ Department of Chemistry, University of Nairobi, Nairobi, Kenya; ^3^ Discipline of Pharmaceutical Sciences, College of Health Sciences, University of KwaZulu-Natal, Durban, South Africa

**Keywords:** photodynamic antimicrobial chemotherapy, photosensitizers, antimicrobial resistance, porphyrins, microorganisms

## Abstract

The reduction of available drugs with effectiveness against microbes is worsening with the current global crisis of antimicrobial resistance. This calls for innovative strategies for combating antimicrobial resistance. Photodynamic Antimicrobial Chemotherapy (PACT) is a relatively new method that utilizes the combined action of light, oxygen, and a photosensitizer to bring about the destruction of microorganisms. This technique has been found to be effective against a wide spectrum of microorganisms, including bacteria, viruses, and fungi. Of greater interest is their ability to destroy resistant strains of microorganisms and in effect help in combating the emergence of antimicrobial resistance. This manuscript reviews porphyrins and porphyrin-type photosensitizers that have been studied in the recent past with a focus on their structure-activity relationship.

## Introduction

Infectious diseases continue to be one of the greatest healthcare challenges worldwide. The burden associated with these diseases remains high with the predominant diseases being tuberculosis, HIV/AIDS, acute lower respiratory tract infections, diarrheal diseases, urinary tract infections, skin and soft tissue infections, infective endocarditis, and sepsis among others (Laxminarayan et al., 2020; Nicholson, 2020). The emergence of antimicrobial resistance (AMR) has further exacerbated the situation. A recent review by O’Neal forecasted that over 10 million deaths will be attributed to AMR by the year 2050 (O’Neill, 2014). Of these infectious diseases, bacterial infections play a significant role, with a high number of deaths worldwide associated with them. Since the launch of antibiotics more than 70 years ago, with the introduction of penicillin, antibiotics have contributed significantly to the decrease in morbidity and mortality rates associated with bacterial infections (Frieri et al., 2017). However, the increasingly rampant antibacterial resistance threatens to send us back to the pre-antibiotic era. Consequently, the WHO has recognized ‘the fight against antimicrobial resistance’ as a global priority that urgently requires newer treatment strategies (WHO, 2018; WHO, 2020).

One of the common denominators to AMR has been the use of conventional antimicrobial agents. These conventional agents have various limitations such as insufficient bacterial concentrations at the site of infections, exposure of healthy tissues and normal flora to the drug, poor adherence to prescribed regimens that require frequent administration, and various undesirable adverse events that have led to the development of bacterial resistance, consequently limiting the success of the treatment regimens (Omolo et al., 2018). The widespread misuse of antibiotics has also resulted in the growing problem of antimicrobial resistance in community and hospital settings (Rice, 2012). Moreover, most antimicrobial classes of antibiotics such as the β-lactams, glycopeptides, and fluoroquinolones have reportedly already developed resistance (Rice, 2012). Furthermore, most of the antimicrobial agents newly introduced to the market are modifications of the existing antimicrobial agents, and they thus lack a new mechanism of action (Theuretzbacher et al., 2020). Therefore, there is a need for a paradigm shift by introducing new agents that have novel mechanisms of action to fight AMR. This review summarizes the available research evidence on the use of porphyrin photosensitizers and their application in Photodynamic Antimicrobial Chemotherapy to eliminate disease-causing microbes. Additionally, the review will focus on structural modifications that have been made on porphyrins and delivery technologies that have been incorporated to further enhance their antimicrobial properties.

## Photodynamic Antimicrobial Chemotherapy

Photodynamic Antimicrobial Chemotherapy (PACT) is a promising strategy to eliminate pathogenic bacteria. Its mechanism of action occurs via the cytotoxic reactive oxygen species (ROS), which are generated by the photosensitive moieties after light irradiation. Upon absorption of light, the photosensitizer is excited to a higher excited singlet state. This is immediately followed by intersystem crossing of the excited photosensitizer to the excited triplet state. The electrons are then quenched by molecular oxygen to generate the toxic ROS, which is responsible for killing the microorganism. (Mai et al., 2017), (Coitiño et al., 2014). The ability of PACT to act on a wide range of bacteria, i.e., gram-negative and gram-positive, antibiotic-sensitive, and multi-resistant strains, presents a tremendous advantage that has made the technique gain a lot of research attention as an alternative strategy to combat antimicrobial resistance (Zeina et al., 2001; Sobotta et al., 2019). To date, various photosensitizers such as phenothiazines, acridines, phthalocyanines chlorins, and porphyrins have been studied for use as PACT photosensitizers (Skwor et al., 2016).

### Porphyrins in PACT

**FIGURE 1:**
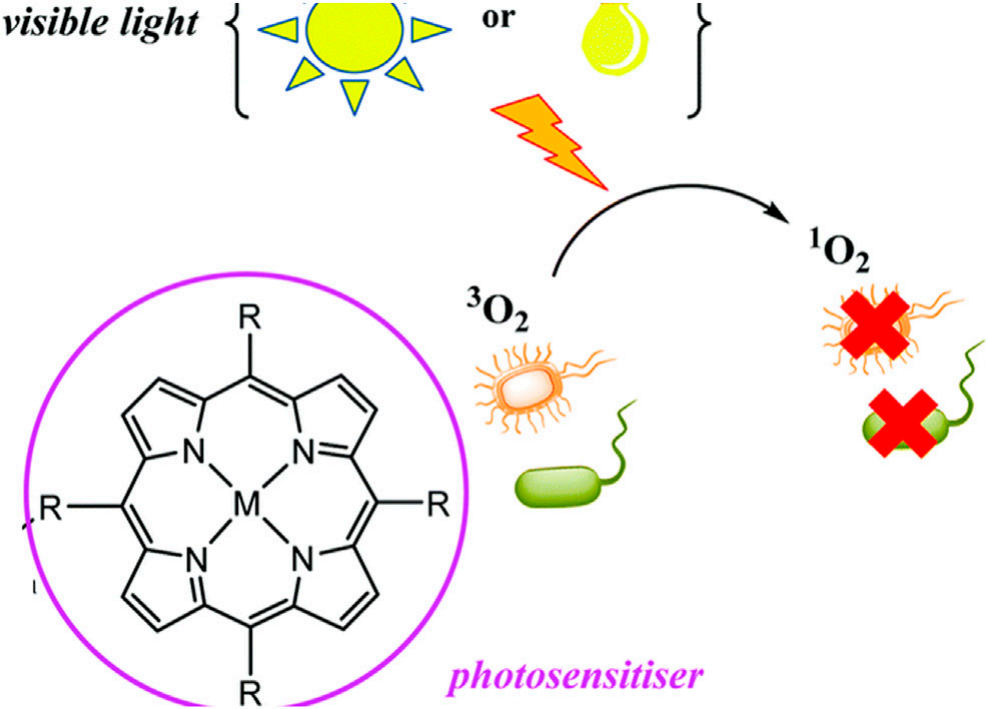
Production of ROS by porphyrins and application in PACT. The illustration shows a generation of singlet oxygen upon irradiation by light which leads to microorganism destruction. Adapted with permission from Spagnul et al. (2017).

Porphyrins and other tetrapyrrole molecules such as phthalocyanines and chlorins possess many desirable properties for use as photosensitizers in PACT. Key among these is their ability to absorb strongly in the UV-Vis near the IR region of the electromagnetic spectrum and their ability to generate a considerable triplet quantum yield, which makes them remarkable generators of ROS (Biscaglia and Gobbo, 2018; Nam et al., 2000) as shown in Figure 1. As a result, porphyrins have been found to have remarkable potential as antimicrobial agents (Vzorov et al., 2002).

### Finetuning Porphyrin-Based Photosensitizers Properties for PACT

**FIGURE 2:**
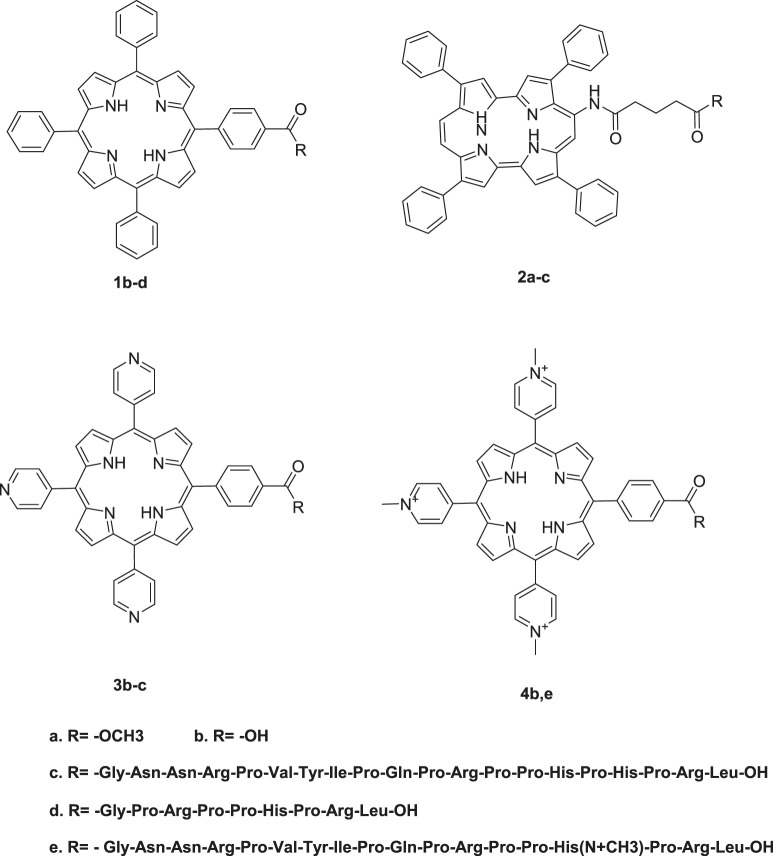
Conjugates obtained from the conjugation of porphyrins with antimicrobial peptides. There was enhanced PACT activity when the conjugates were used. By the use of the amide linkage, different peptides with different amino acid sequences and lengths were couped to the free porphyrins. The hydrophilicity offered by peptides together with the hydrophobicity of the peptides resulted in a conjugate that was amphophilic. The amphiphilicity results in better solubility of the free porphyrins Reproduced with permission from Dosselli et al. (2013).

**FIGURE 3:**
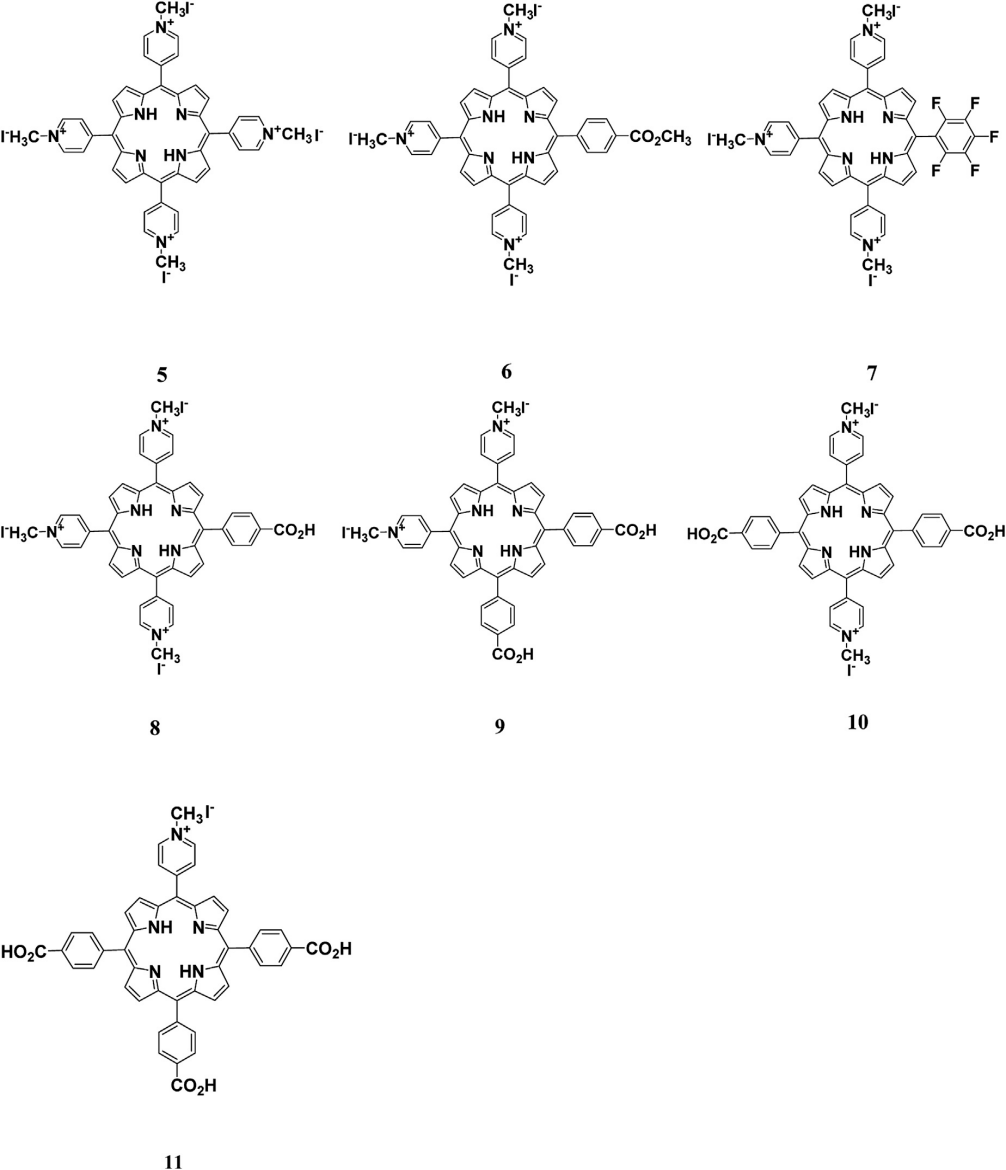
Cationic porphyrin derivatives employed for photoinactivation of bacteria. Quaternization of the porphyrins resulted in systems with high positive charge density. Highly cationic systems were found to have enhanced antimicrobial activity. The positive charge was found to enhance the binding of the system to negatively charged bacteria leading to bacteria destruction. Reproduced with permission from Alves et al. (2009).

**FIGURE 4:**
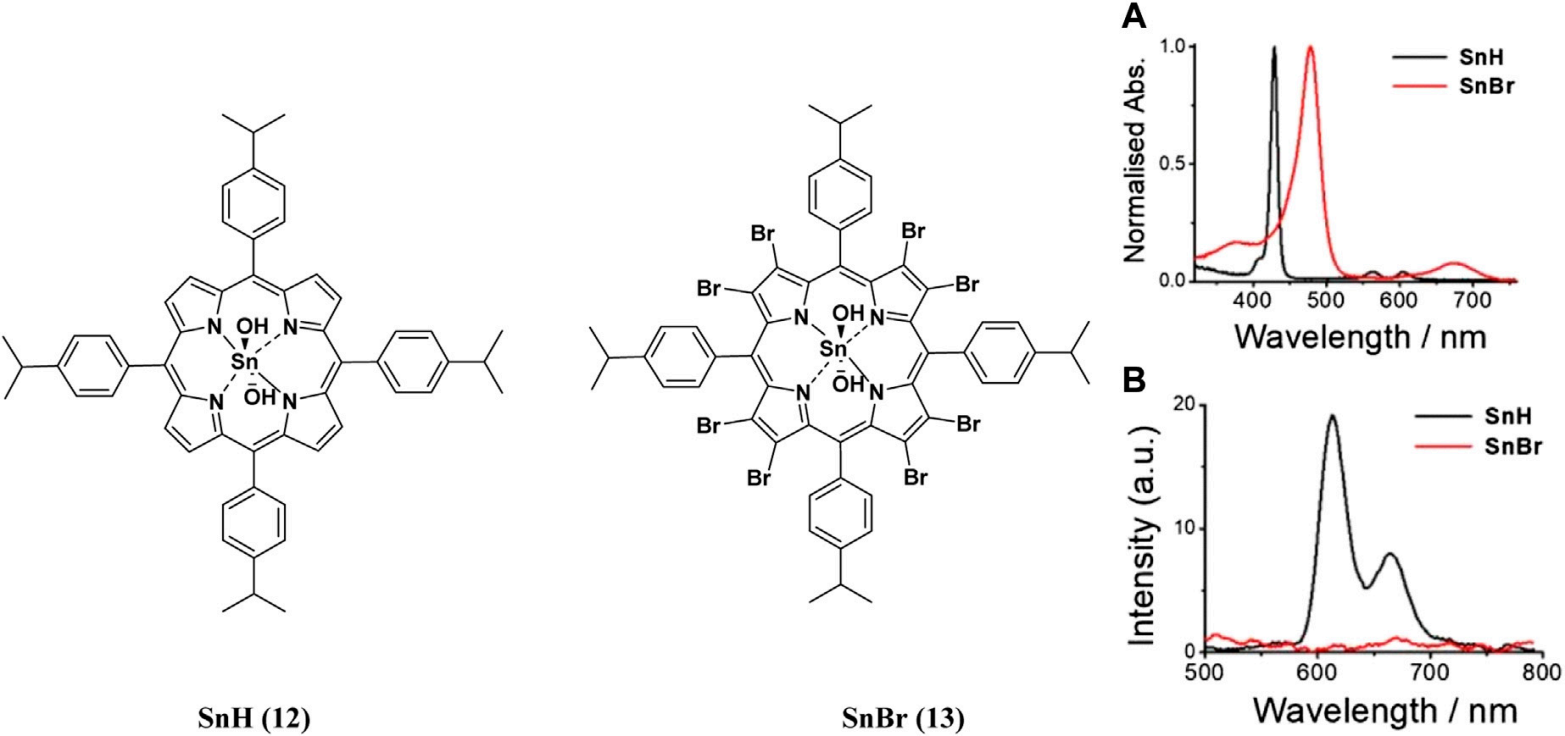
**(A)** UV-Vis absorption **(B)** and emission spectra of porphyrins 12 and 13. These indicate the redshift in wavelength of absorption and quenching of the fluorescence upon bromination of the porphyrin. Reproduced with permission from porphyrin. Reproduced with permission from (Babu et al., 2020a).

Porphyrins have a very flexible structure that can be modified in different ways to improve their photophysical and biological properties. This can be achieved through the insertion of different metals in the porphyrin's core or by selectively changing the peripheral substituents attached to the porphyrin skeleton at the meso, β-positions, or the porphyrin core (Beirão et al., 2014; Prasanth et al., 2014; Zoltan et al., 2015). Moreover, porphyrins have been reported to have very low bioavailability, a property that is attributed to their poor water solubility due to their hydrophobicity. Therefore, modifying their structures by adding polar substituents or by conjugating them with hydrophilic moieties such as amino acids, peptides, and proteins can lead to improved water solubility, which is an important property for their application in PACT (Figures 2–4).

As illustrated in Table 1, free porphyrins show potential for PACT application. Using various techniques that involve either complexation or covalent conjugations, various biomaterials have been employed in the modification of the physico-chemical and pharmacological properties of the attached porphyrin. For example, metals such as titanium dioxide (TiO_2_) have been complexed with meso-tetrakis (p-sulfonatophenyl) porphyrin, and the resulting complexes showed improved photostability, fluorescence, and self-assembly into nanoparticles (Sułek et al., 2019). Another approach has involved the conjugation with amino acids, which added hydrophilicity and overall positive charge of the system. Conjugation with antimicrobial peptides (AMPs) and cell-penetrating peptides (CPPs) has also been reported. The use of AMPs and CPPs has led to an extended spectrum of activity, photostability, antimicrobial properties in the presence or absence of light, and the ability of the system to penetrate the cells and targeted organs (Dosselli et al., 2010). Other biomaterials also commonly used for fine‐tuning the porphyrins are fatty acids. As shown in Table 1, fatty acids such as oleic acid and palmitic acid have been used to make superior porphyrin-based PACT systems. Such systems have exhibited better membrane penetration ability, enhanced microbial activity, reduction in aggregation of porphyrins, and high single oxygen production (Babu et al., 2019; Babu et al., 2020b). These modifications and the observed improvements are summarized in Table 1.

**TABLE 1:** Porphyrin fine tuning and the resulting conjugate properties.

Type of porphyrins	Material used in modification	Effect of modification	References
5,10,15,20-tetrakis-(4-sulfonatophenyl)porphyrin (TPPS); 5,10,15,20-tetrakis(2,6-difluoro-3-sulfophenyl)porphyrin (F2POH), and 5,10,15,20-tetrakis(2,6-difluoro-3-sulfophenyl)porphyrin Zn(II) (ZnF2POH)	titanium dioxide (qTiO_2_)	Stability of the resulting nanoparticles	Sułek et al. (2019)
		Increase in the fluorescence when compared to free Porphyrins	
		Increased levels of ROS generation after impregnation of qTiO2	
		Multiple mechanisms of ROS generation	
		Exhibited antimicrobial activity at a very low concentration	
		Broad‐spectrum of activity	
5-(4-nitrophenyl)-10,15,20-tripyridylporphyrin	Filter paper (cellulose) and cyanuric chloride as the linking agent	A strong photobactericidal effect against *S. aureus* and *E. coli.*	Mbakidi et al. (2013)
5,10,15,20-Tetrakis(4-N-methylpyridyl)-21H,23H-porphyrin.	Polymyxin B	Synergistic effect of Polymyxin B and PACT	Le Guern et al. (2017)
		Increased uptake by Fibroblasts thus increasing wound healing.	
		Expanded spectrum of activity of Polymyxin B to gram-positive bacteria after conjugation.	
Nitrotetraphenylporphyrin	amino acids, l-lysine, l-histidine, and l-arginine,	Amino acid conjugation resulted in water solubility	Meng et al. (2015)
		Increased photostabilities	
		Increasing conjugation with lysine increased production of singlet oxygen species	
		Better photoinactivation abilities of bacteria when compared to the free Porphyrins	
		Conjugates were resistant to degradation in serum within 24 h.	
		Good biocompatibility	
2-hydroxypyridine axial ligated indium 5,10,15,20-tetrakis-(4-phenylmethylthio) porphyrin (3) and quaternized 2-hydroxypyridine axial ligated indium 5,10,15,20-tetrakis-(4-phenylmethylthio) porphyrin	oleyamine and oleic acid (OLA)	8 log reduction in bacteria	Collen Makola et al. (2021)
		100% bacteria elimination after 25 min irradiation	
tetrakis(N-methylpyridyl)porphyrin (TMPyP)	Lysine Analogue of Polymyxin B	4 log reduction compared to the untreated control)	Le Guern et al. (2018)
		Photobactericidal activity against Gram-positive as well as Gram-negative bacteria	
Tetrakis(4-carboxyphenyl) porphyrin (TCPP)	DNA	High ROS generation efficiency and photostability	Kumari et al. (2017)
		Improved killing efficiency of gram-positive *S. aureus* bacteria	
5(4′-carboxyphenyl)-10,15,20-triphenylporphyrin (cTPP)	cationic antimicrobial peptide, apidaecin Ib	Increased water solubility	Dosselli et al. 2010
		Broad spectrum of activity	
		Improved antibacterial activity when compared to the free porphyrin	
tricationic porphyrin [(5,10,15‐tris(1‐methylpyridinium‐4‐yl)‐20‐(pentafluorophenyl)porphyrin triiodide, Tri‐Py+‐Me‐PF] Sn(IV) porphyrins	1‐palmitoyl‐2‐oleoyl‐sn‐glycero‐3‐phosphatidylethanolamine pyridyloxyl trans-axial ligand	Improved antimicrobial effects and hence broad-spectrum coverage against drug-resistant strains of bacteria	Alves et al. (2013)
		Reduction in the aggregation of the porphyrins	Babu et al. (2019), Babu et al. (2020b)
		High singlet oxygen production	
		High killing efficacy against *Staphylococcus aureus*	

## Advancement of Porphyrin-Based Photosensitizers for Targeted Antimicrobial Photodynamic Therapy

Recent advancements in synthetic chemistry and material science have resulted in the development of programable systems that respond to physiological changes due to diseases. PACT has followed a similar trend with the fabrication of systems that are stimuli-responsive to target bacteria. This section will focus on the advancement of porphyrin-based PACT systems for targeting bacteria and bacterial infection sites.

### Porphyrin-Based Photosensitizer Nanosystems

The conjugation of porphyrins to nanoparticles has been explored. The conjugates have been found to exhibit enhanced antimicrobial properties and this is attributed to their biocompatibility and synergistic properties for PACT (Shabangu et al., 2020). By taking advantage of the small size of the porphyrins and porphyrin-nanoparticle conjugates, the photosensitizers can attach to the bacterial cell wall through a self-assembly process, resulting in cell death. In some cases, however, these conjugations have led to unexpected interactions such as reduced uptake by the cells and reduced antimicrobial activity. (Wei et al., 2015; Kashef et al., 2017). The following sections will discuss various porphyrin-based nano-formulations.

#### Self-Assembled Porphyrin-Based Photosensitizers

**FIGURE 5:**
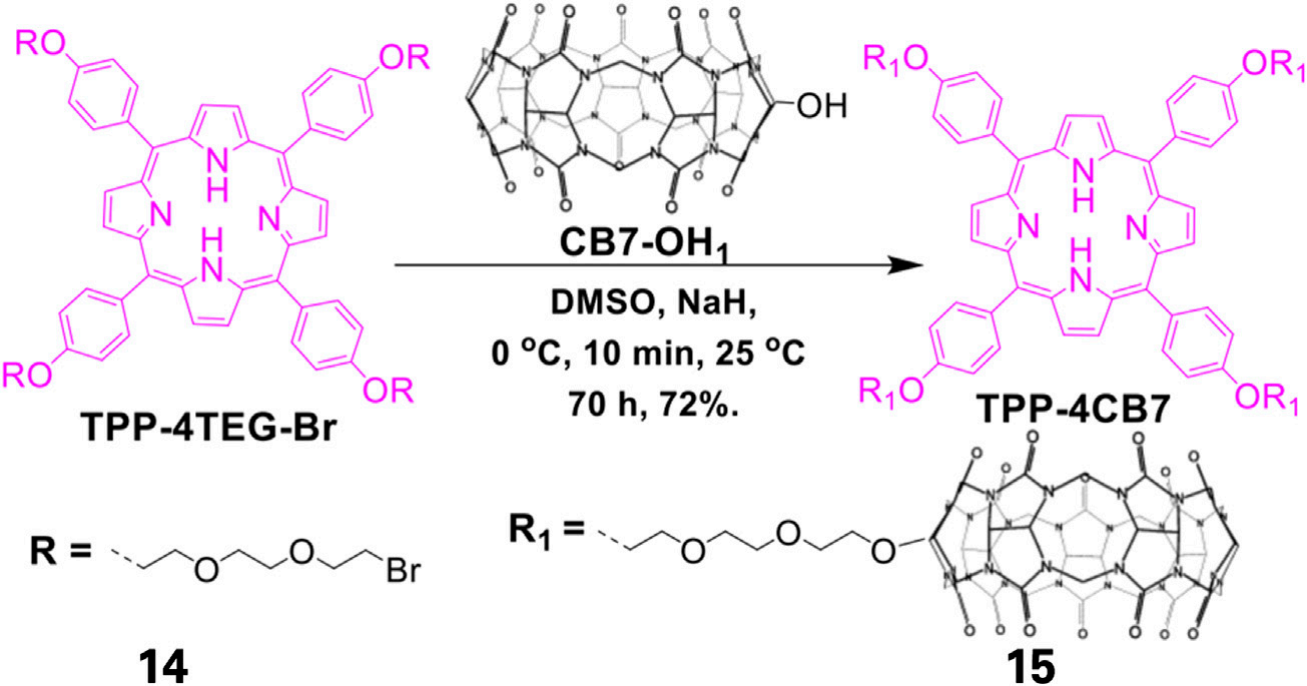
Synthesis of Cucurbit[7]uril-linked Porphyrin-based complex. The free TPP porphyrin was first conjugated to hydrophilic polyethylene glycol after which the resulting conjugate was complexed with that of Cucurbit[7]uril. This reaction resulted in a self-assembling amphiphilic system. Adapted with permission from Özkan et al. (2019).

Formulation of self-assembling photosensitizers has recently become a focus of interest in the field of photodynamic therapy with the synthesis of self-assembled porphyrin-based photosensitizers (SAPPs). SAPPs are synthesized by conjugating hydrophobic porphyrins to hydrophilic or amphiphilic biomaterials such as polymers via covalent or supramolecular conjugations. These conjugations result in self-assembled nanostructures such as micelles (Spiller et al., 1998), polymersomes (Lanzilotto et al., 2018), honeycombs (Wang et al., 2014), nanofibers (Wang Q. et al., 2018), and metal-organic frameworks (MOFs) (Zhu et al., 2020). Electron spin-resonance spectroscopy (ESR) studies have shown that self-assembled porphyrins generate high oxygen singlets that are extremely effective for Photodynamic Therapy (Wang D. et al., 2018). Using supramolecular chemistry, Özkan and co-workers synthesized SAPPs from cucurbit (Wang Q. et al., 2018) uril and porphyrin to form a multifunctional system. The system was found to efficiently eliminate broad-spectrum bacteria via a light-trigger. To further potentiate the antibacterial activity, the system could be loaded effectively with drug molecules. As illustrated in Figure 5 **,** the system was synthesized by conjugation of cucurbit (Omolo et al., 2018) uril shell, which acted as host for loaded drugs, to a free-base porphyrin core via suitable linkers (Kumar et al., 2019; Özkan et al., 2019). While the resulting system exhibited no dark activity towards *E. coli* (gram-negative bacteria), it showed relatively high cytotoxicity on *B. subtilis* (gram-positive bacteria). However, upon exposure to light, the self-assembled system had 100% bacteria elimination for both *E. coli* and *B. subtilis*. Similar SAPPs have been reported, with the systems showing improved PACT activities compared to the free porphyrins (Liu et al., 2013; Li et al., 2018; Khan et al., 2019).

#### Dendrimer Based Porphyrin Photosensitizers

**FIGURE 6:**
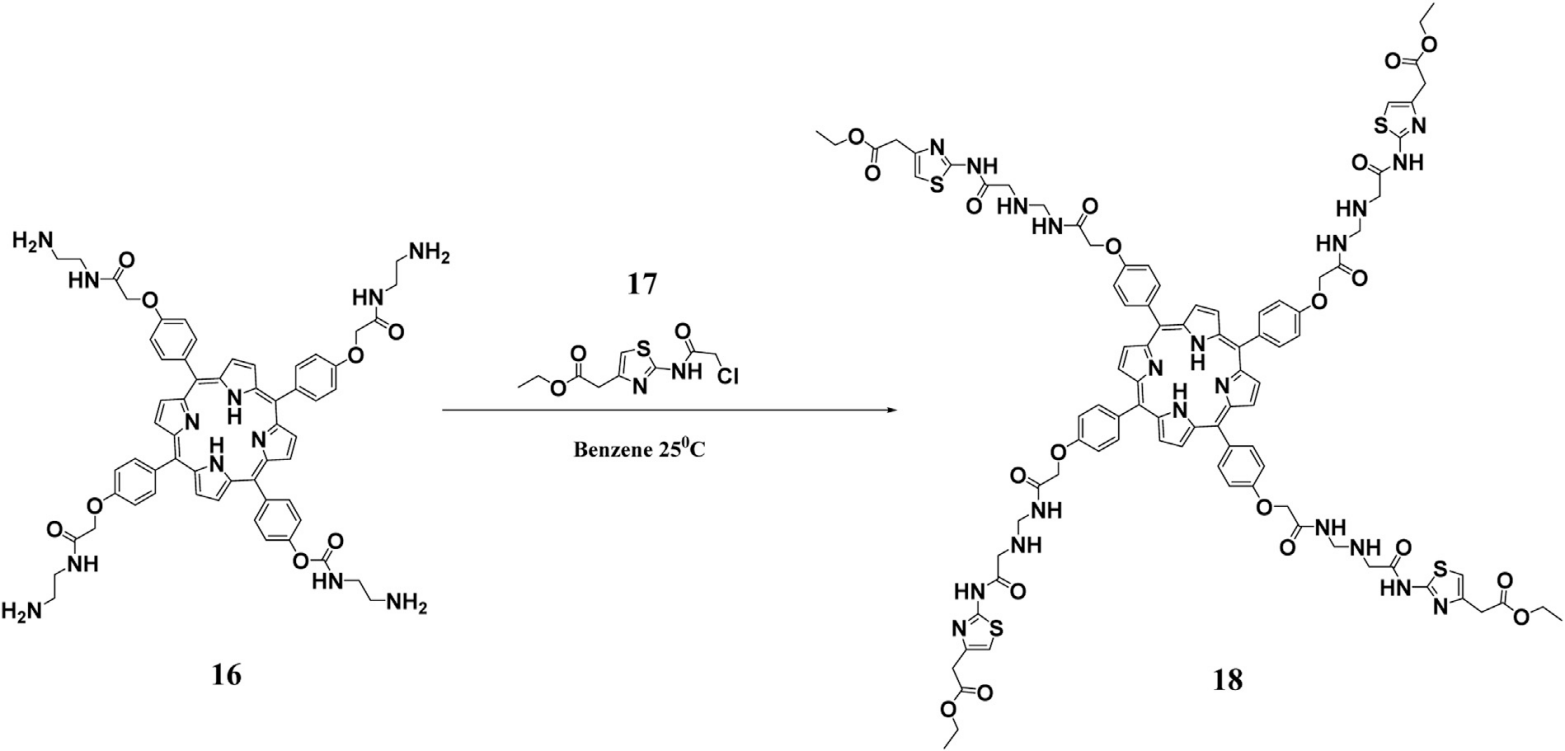
Synthesis of hydrophilic DPs. This scheme shows the reaction of a polyamidoamine (PAMAM) functionalized porphyrin with ethyl 2-(2-chloroacetamido)-4-thiazoleacetate to obtain the hydrophylic DP. Reproduced with permission from Hernández Ramírez et al. (2020).

Given that porphyrins are hydrophobic and have large π conjugation domains, they usually exhibit aggregation which ultimately affects their photo functionalities. This disadvantage can be overcome by Dendrimer porphyrins (DPs) (Gerhardt et al., 2003). DPs have unique photo functional properties including large absorption surface area, increased fluorescence emission, and enhanced photosensitizing properties (Wirotius et al., 2013). DPs remain soluble in aqueous media as a result of the large number of anionic functional groups on their periphery, which arise from the dendrimer conjugations **(** Figure 6 **).** Moreover, they have wedges that effectively prevent aggregation. Studies have shown that DPs have about 10–100-fold higher photosensitizing effects when compared to bare protoporphyrin systems (Zhang et al., 2003). Penon and co-workers constructed a DP superstructure by coating the surface of the iron nanoparticles with two modified protoporphyrin molecules. From the study, it was found that DP with tris(hydroxymethyl)aminomethane (TRIS) modified protoporphyrin had two-fold singlet oxygen production ability when compared to the hydrophobic porphyrin system. It was concluded that hydrophilic systems were better for photodynamic therapy than hydrophobic ones (Penon et al., 2016). In another study, Staegemann and co-workers synthesized a high molecular dendritic mannose‐functionalized hyperbranched polyglycerol and loaded it to a zinc porphyrin. The hydrophobic zinc (II) porphyrin photosensitizer was solubilized when it was loaded in the mannose- polyglycerol system. Further studies showed that an increased number of mannose molecules resulted in increased solubility of the zinc porphyrin, which led to better photosensitivity and consequently enhanced antibacterial activity (Staegemann et al., 2017). The increased photosensitivity and improved antimicrobial activity has been attributed to properties such as multivalence, an increased surface area to volume ratio, and an increased solubility that dendrimers have (Omolo et al., 2018).

#### Liposomes Based Porphyrin Photosensitizers

Liposomes are excellent vehicles for intracellular delivery as they easily fuse with biological membranes (Omolo et al., 2019). Ferro and co-workers explored the positive traits of liposomes for intracellular delivery of two photosensitizing agents, hematoporphyrin and chlorophyll, for the elimination of Methicillin-resistant *Staphylococcus aureus* (MRSA) (Ferro et al., 2006). From the study, when loaded with hematoporphyrin, the liposome led to improved endocellular absorption of the photosensitizer compared to when the cells were incubated with the free porphyrin. The hematoporphyrin-loaded liposome also displayed improved binding and more efficient photoinactivation of MRSA. Interestingly, hematoporphyrin did not affect the three-dimensional organization of the liposome during the photoinactivation of MRSA. On the other hand, chlorophyll markedly destroyed the structure of the lipid vesicle with no visible phototoxicity to MRSA. The same research group synthesized a positively charged *meso*-substituted porphyrin, (5-[4-(1-dodecanoylpyridinium)]-10,15,20-triphenyl-porphyrin) and delivered it *via* cationic liposomes for the elimination of MRSA. The free porphyrin had an unusually large fluorescence quantum yield (0.95), which led to the limited generation of singlet oxygen (Ferro et al., 2007). The porphyrin displayed a relatively low photosensitizing activity against MRSA when dissolved in an aqueous solution or when incorporated into neutral liposomes. However, when the porphyrin was loaded to a cationic liposome, the phototoxicity effects against the bacteria increased remarkably. The increased effect upon loading the synthesized porphyrin in the cationic delivery system was attributed to the increased positive charge density (Hassan et al., 2020) that destroyed the bacterial wall, thereby enhancing the permeability of the photosensitizer. A similar study was reported by Bombelli and co-workers (Bombelli et al., 2008). Moreover, high cationic charge density often has non-selective toxicity even to human cells (Fischer et al., 2003). Despite the good antimicrobial results, the study did not perform any cytotoxicity studies, and the application of the system on biotic systems will therefore be questionable.

#### Other Nano Based Porphyrin Photosensitizers

PACT has drawn the interest of nanotechnology as the efficacy of the treatment can be greatly augmented using nanoparticles. Nano-based porphyrin photosensitizers can be morphed into various nanosystems. One of the applications of the nanosystems is the improvement in the delivery of a photosensitizer to the bacteria and fluorescence inactivation kinetics. Nanosystems such as polymeric nanoparticles have been loaded with porphyrins to enhance the delivery to microorganisms and improve PACT activity. The polymeric nanosystems in focus have been on systems coined from biocompatible and biodegradable polymers such as from polylactic glycolic acid (PLGA) (Mai et al., 2020), poly(ε-caprolactone) (PCL) (Liu et al., 2017; Kubát et al., 2019; Chen et al., 2020; Contreras et al., 2020), gelatin (Kirar et al., 2019), and cyclodextrins (Ferro et al., 2009; Castriciano et al., 2017; Khurana et al., 2019; Zagami et al., 2020). Several reports of many nanosystems for PACT have been reported with great success as shown in Table 2 **.**

**TABLE 2:** Different nanosystems for delivery of photosensitizers.

Porphyrin used	Biomaterials	Nanocarrier	Irradaiation conditions	Bacteria tested on	Activity	Significant findings	References
4-(15-(4-(2-carboxyethyl)phenyl)porphyrin-5-yl)-1-methylpyridin-1-ium Iodide	Gelatin	phototheranostic polymeric nanoparticle	Green LED (0.5W, 520–560 nm) for about 3 h	*Escherichia coli, Serratia marcescens, Pseudomonas putida, Bacillus subtilis, Candida viswanathii*	5 and 6 log antimicrobial activity translating to about 99.999% elimination	Excellent hydrophilicity, biocompatibility, and stability,	Kirar et al. (2019)
						High ^1^O_2_ quantum yield (44%),	
						High fluorescence quantum yield (69%)	
						Elimination of up 99.9999% of the gram-negative and positive bacteria and fungus.	
Hematoporphyrin (HP) and Chlorophyll a (Chlorin)	Cationic lipids	Liposomes	White light from a Teclas Lamp, 100 mW/cm^2^ For 30 min	MRSA	>5 log inhibition of MRSA by chlorin alone in 10 min. There was, however, a reduction in activity when the delivery systems were applied For HP, the delivery system greatly enhanced the inhibition activity	Endocellular concentration of photosensitizer	Ferro et al. (2006)
						Elimination of MRSA	
zinc meso-tetra (4-pyridyl) porphyrin (ZnTPyP)	Zinc *meso*-tetra (4-pyridyl) porphyrin	Cubic nanoparticles	Solar simulator for 120 min	*E. coli*	50% was eliminated after 30 min. By 120 min, all the *E. Coli* was completely eliminated	Synthesized porphyrin self-assembled into cubic nanoparticles	Wang D. et al. (2018)
						High singlet oxygen quantum yields	
						Fairly stable for a long time in dark at ambient conditions. Attractive property for storage and transportation.	
						Effective elimination of bacteria	
Sinoporphyrin sodium (DVDMS)	PLGA	Nanohybrids	Different visible laser doses	*Staphylococcus aureus* and multidrug-resistant (MDR) *S. Aureus*	4-log (99.9918%) inactivation of MRSA and 5-log (99.9995%) inactivation of *S. aureus*	Eliminated *Staphylococcus aureus* and multidrug-resistant (MDR) *S. Aureus*	Mai et al. (2020)
						Accelerated wound healing in a burn infection model.	
						Increased several regenerative factors.	
						Fluorescence imaging achieved	
Tetrakis(4-carboxyphenyl)porphyrin	Bimetallic PCN-224(Zr/Ti)	Metal–Organic Framework	Visible light (200 mW cm^−2^) for 3 min	*S. aureus, S. epidermidis, E. coli, A. baumannii,* MRSA, MRSE, MDR *E. coli and* MDR *A. baumannii*	96.4% MDR E. coli, 96.8% MRSA, and 96.2% MRSE were eliminated	Elimination of multidrug-resistant bacteria	Chen et al. (2020)
						High singlet oxygen quantum yields	
						Accelerated wound healing	

Table 2 summarizes the improvements in the physicochemical and pharmacological properties of PACT systems when porphyrins are incorporated in nanocarriers. Different porphyrins have been incorporated in various nanosystems, such as liposomes, cubosomes, nanohybrids, and metal organic frameworks (MOF), to form multifunctional systems. For example, the combination of the porphyrins with biomaterials has resulted in systems that can be employed for theranostic purposes and PACT (Kirar et al., 2019). The porphyrin-based nanoformulations have also been reported to have controlled release and distribution properties for the singlet oxygen species and enhanced absorption in targeted cells and organs.

### Multifunctional Porphyrin Based Systems

**FIGURE 7:**
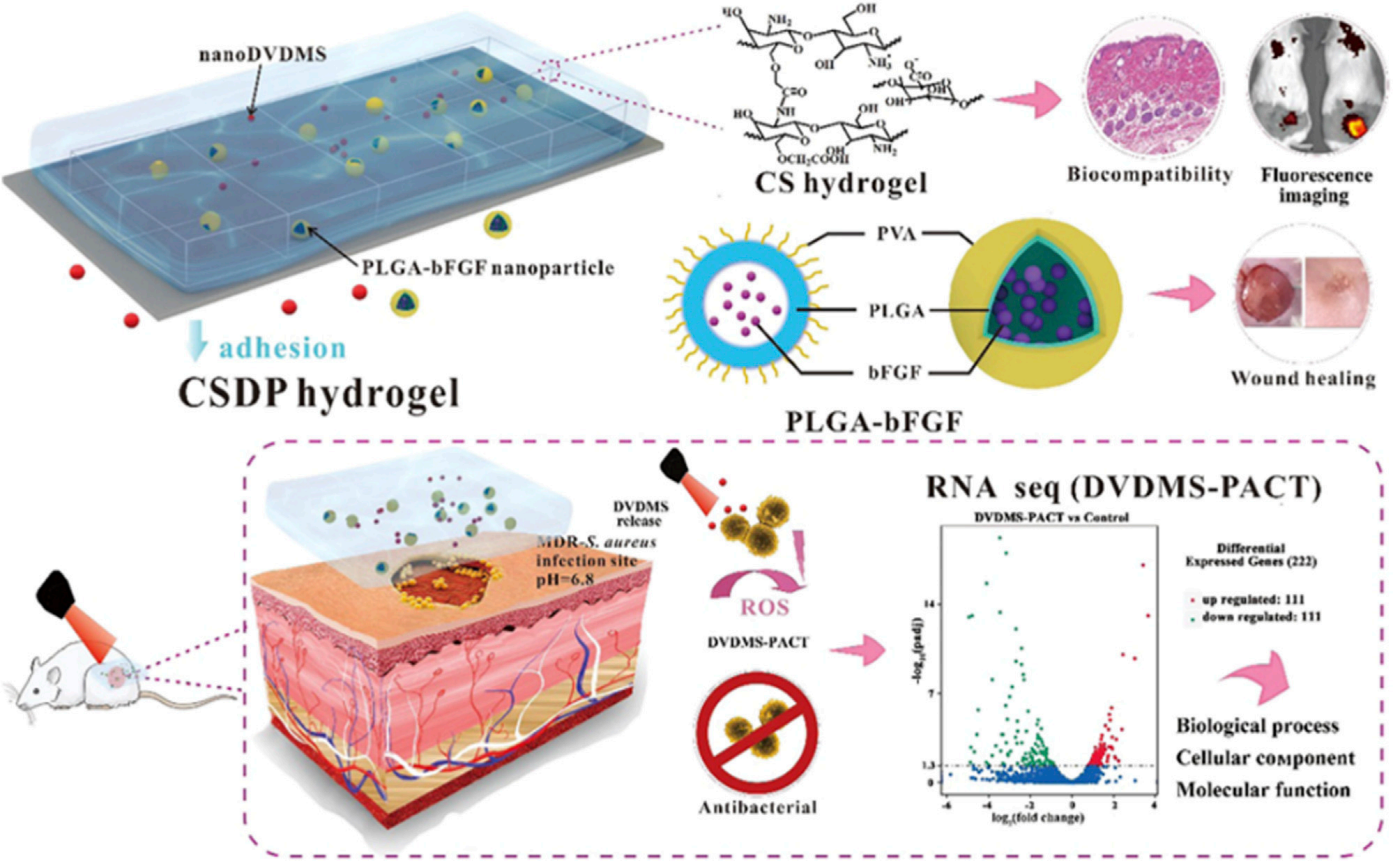
Schematic representation of the multifunctionalities of the hydrogel; with improved bioavailability, there’s improved reduction in bacteria and improved wound healing. Adapted with permission from Mai et al. (2020).

**FIGURE 8:**
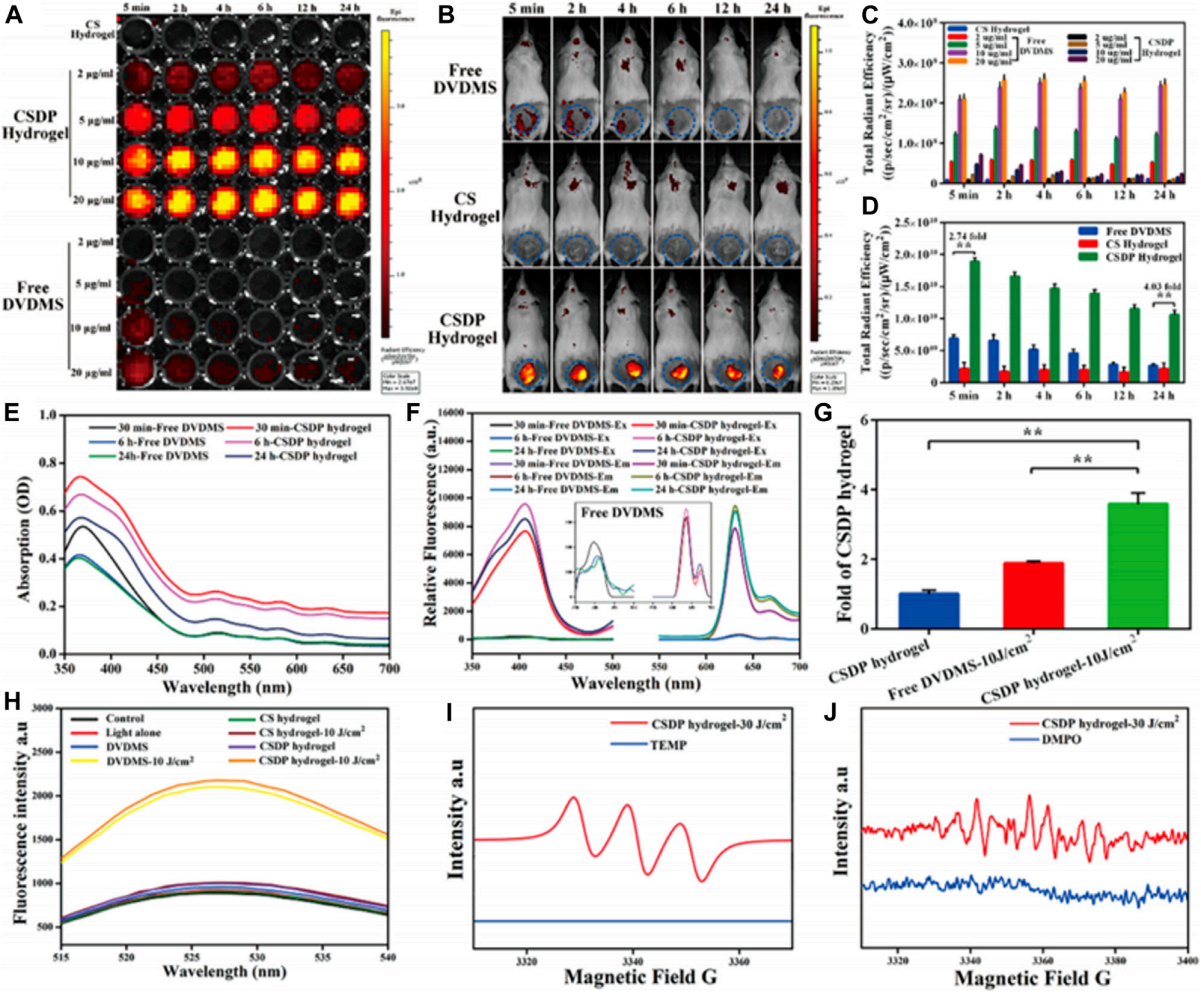
Fluorescence imaging of the hydrogel via 96 well-plates **(A)** and *in vivo* mice models **(B)**. The figures showed the emittance of fluorescence from the hydrogel loaded with an imaging agent and PACT. The *in vitro* results were reproduced in tests done in animals through the mice model showing the translational potential of the system. **(C**,**D)** quantitative analysis of the fluorescence imaging *in vitro* and *in vivo*, respectively. Absorbance **(E)** and fluorescence spectra **(F)** of nanosysten, hydrogel loaded with the nanospheres. **(G)** Singlet oxygen yields of hydrogel, free nanospheres, and hydrogel-loaded nanospheres as determined *via* a fluorescence spectrophotometer. **(H)** SOSG spectra of different groups. **(I**,**J)** Detection of free radicals via ESR spectra and single oxygen species generation from the hydrogel in the dark and light irradiation, respectively. From the results, the hydrogel formulation was determined to be superior to the free systems. Reproduced with permission from Mai et al. (2020).

The advancement of synthetic chemistry and material science has resulted in the development of various multifunctional porphyrin-based systems. These include systems such as theranostic, wound-healing, and antimicrobial systems that have been reported in literature. Mai and co-workers reported a multifunctional porphyrin loaded nanosystem that was employed in the treatment of burn infections, stimulation of wound healing, elimination of a wide spectrum of bacterial via PACT, and bioimaging **(** Figure 7 **)** The system was composed of the porphyrin photosensitizer, sinoporphyrin sodium (DVDMS), and poly(lactic-co-glycolic acid) (PLGA) was encapsulated with basic fibroblast growth factor (bFGF) and formed nanospheres. The nanospheres were implanted in a carboxymethyl chitosan (CMCS)–sodium alginate hybrid hydrogel. The system was evaluated for antibacterial properties against multidrug resistance bacteria (MDR), rheological properties, fluorescence imaging, and biocompatibility. The results indicated that the system had a 99.99% elimination of *S. aureus* and MDR *S. aureus* in mice models. Moreover, the nanosystem exhibited enhanced wound healing and regulation of regenerative and proinflammatory factors (Figure 8) (Mai et al., 2020). Another multifunctional porphyrin system was reported by Dai and co-workers. Their system was a thermosensitive and photosensitive micelle that was formulated from a star polymer, poly(ε-caprolactone)-block-Poly(*N*-isopropylacrylamide), which had a porphyrin-core (Dai et al., 2014). The system showed potential for multifunctionality and application. However, further characterization of the system is needed. Other multi-functional porphyrin-based systems, with antimicrobial and cancer therapy applications, have been reported in the literature (Belali et al., 2017; Cao-Milán et al., 2020; Li et al., 2020).

## *In Vivo* Applications of PACT Based on Porphyrins

*In vivo* studies are paramount to evaluate the translational ability of the formulated system to human trials. PACT systems have been gaining momentum as a treatment. The possible evaluation of clinical applications of PACT has included treatment of wound infections, body cavity infections (such as the mouth, nasal sinuses, and ear), and surface infections of the skin and cornea. This section will evaluate the various application of PACT systems in animal models.

### PACT in Treatment of Wounds and Acceleration of Wound Healing

The effects of PACT on the treatment of skin infections have mostly been demonstrated in *in vitro* studies. What is still lacking is a complete *in vivo* evaluation of the feasibility of PACT in treating skin and soft-tissue infections. Many researchers have in recent times discouraged the over-reliance on *in vitro* studies results in PACT because of the variations of outcomes encountered when carrying out the complementary *in vivo* studies. This means that conducting *in vivo* follow-up studies, right after an *in vitro* study, is key in establishing the overall efficacy of PACT.

*In vivo* studies were carried out by Fila and co-workers where they utilized four photosensitizers in a murine model with chronic wounds that were infected with *Pseudomonas aeruginosa* and MRSA. While comparing the *in vivo* results with their past *in vitro* findings, they observed that there was a considerable reduction of the effectiveness of the therapy *in vivo*. The photosensitizers, 5,10,15,20-tetrakis(1-methyl pyridinium-4-yl)porphyrin (TMPyP), Rose Bengal, [Ru(2,2′ -bipyridine)2(2-(2′,2″:5″,2‴-terthiophene)-imidazo[4,5-f][1,10] phenanthroline)]2+ (TLD-1411), and methylene blue demonstrated good antimicrobial efficacy in 1–50 μM planktonic solutions; however, in *in-vivo,* there was a 24–48 h growth delay for MRSA and an extended growth inhibition of *P. aeruginosa* by the TLD-1411-assisted photodynamic therapy. In the *in vitro* studies, a 6 log_10_ reduction was attained even with the least concentration of 1 mM. However, in the *in vivo* tests, none of the photosensitizers achieved sterilization that was even equivalent to 3 log_10_ reduction based on bioluminescence radiance measurement (Fila et al., 2016).

Nonetheless, completely different observations have been made in other studies with better outcomes emerging during *in vivo* studies. For instance, Xu and co-workers (2016) studied the wound healing effect of PACT on mixed bacterial infections in rats using the lysin conjugate of 5,10,15,20-tetrakis(1-methyl pyridinium-4-yl)porphyrin tetra-iodide. Their studies demonstrated that the porphyrin was highly potent both *in vitro* and *in vivo*. It was also observed that the applied dose of light was a key factor for the success of PACT. In this study, a light dose of 50 J/cm^2^ was established as the most suitable (Xu et al., 2016). A similar study done by Yuan and co-workers (Yuan et al., 2017) found that the cationic lysine-porphyrin conjugate (**12**) accelerated wound healing, while the cytotoxicity test conducted in mice showed that the porphyrin was not toxic.

**FIGURE 9:**
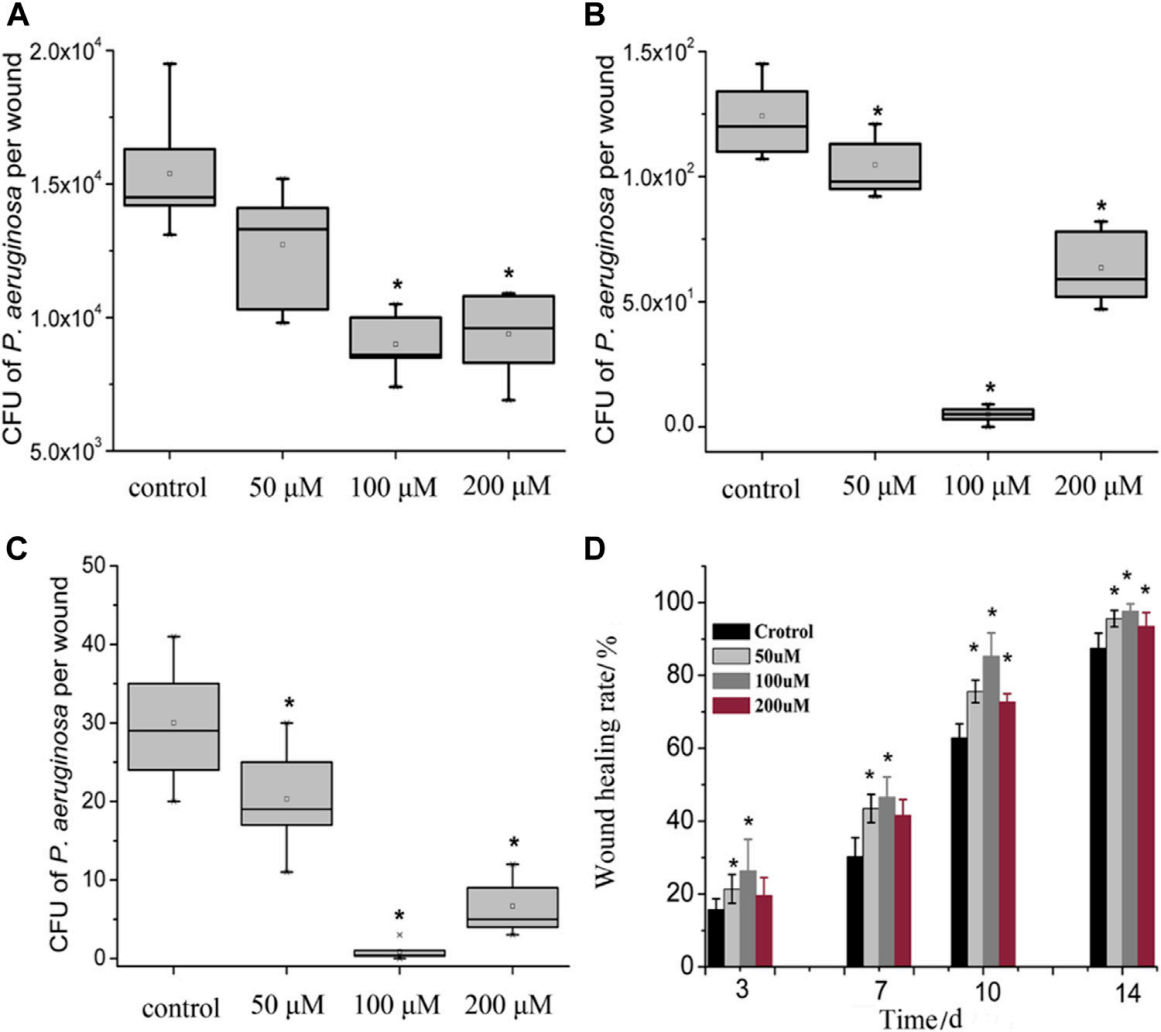
Box plot of the viability of bacteria in wounded tissue in. Wound healing rate in the PACT chemotherapy groups when compared control groups on evaluated at different days. **(A–C)** showed there was a dose-dependent reduction of the CFU recovered from the wounds in various treatment groups when compared to the control. Treatment groups receiving 100 µM showed the best antimicrobial activity. **(D)** showed that the healing percentage correlated to the reduction of CFUs illustrated in **(A–C)**. A reduction in CFU recovered was accompanied by an increase in wound healing percentage. Reproduced with permission from Zhao et al. (2020).

The effectiveness of PACT in treating infected wounds has also been recently studied by Zhao and co-workers using a protoporphyrin IX–ethylenediamine derivative **(13)** against *Pseudomonas aeruginosa* in an *in vivo* model of *P. aeruginosa*-infected wounds. Their study showed a significant reduction of the number of *P. aeruginosa* colonies. Additionally, the histological analysis demonstrated a very high wound healing rate (98%) after 14 days of therapy (Figure 9). According to the findings, 100 μM concentration of the porphyrin resulted in a 4.2 log_10_ reduction of *P. aeruginosa* colony units, which translated to about 10% more activity compared to the control group (Zhao et al., 2020).

### PACT in Treatment of Body Cavity Infections

*In vivo* studies on the effects of PACT in oral fungal infections commonly caused by *C. albicans* were studied by Mima and co-workers. The group used Photogene (hematoporphyrin derivative) and two light sources—blue (455 nm) and red (630 nm)—to carry out their studies. From their results, there was a significant reduction in *C. albicans* obtained from the tongues of mice. The results from the histological evaluation showed that the local mucosa was not adversely affected by PACT (Mima et al., 2010). In a study by de Santi and co-workers (2018), PACT was also found to be effective against yeast cells that cause vaginal candidiasis. Utilizing protoporphyrin IX, among other photosensitizers, there was a significantly reduced *C. albicans* population, which was accompanied by prevention of further re-infection for about 1 week (de Santi et al., 2018). The results of these studies show alternative treatment of fungal infections by PACT is not only feasible but also safe.

Researchers have in the recent past explored various treatment methods including the effectiveness of PACT in the treatment of periodontitis (Moreira et al., 2015; Stájer et al., 2020). For instance, Prasanth and co-workers synthesized pyridinium-substituted porphyrin derivative **14** and *meso-*imidazolium-substituted porphyrin derivative **15** and studied their activity. The derivatives not only showed complete penetration into biofilms but also displayed better efficacy against the oral pathogens associated with periodontitis such as *F. nucleatum, E. faecalis*, and *A. actinomycetemcomitans* (Prasanth et al., 2014)*.*

With most studies focusing on *in vitro* evaluation, an *in vivo* study was successfully carried out by Sigusch and co-workers using the beagle dog model. From this study, they observed that photodynamic therapy using chlorine e6 and 662 nm laser light source resulted in significant suppression of *P. gingivalis* and there was an overall reduction of the periodontal signs of redness and bleeding on probing (Sigusch et al., 2005). To experiment on human models, a full-mouth PACT in *F.nucleatum*-infected patients was carried out. The study established that the adjuvant application PACT method was effective in reducing periodontal inflammatory symptoms and treatment of *F. nucleatum* (Sigusch et al., 2010).

## Future Perspectives

As we have described in this review, porphyrins and their use in PACT continue to draw a lot of attention, with novel porphyrins and porphyrin conjugates being continuously synthesized. Indeed, the possibilities are unlimited. With the development of many different porphyrin synthetic routes coupled with their flexibility for modifications and conjugations to different moieties, more superior porphyrins can be designed and synthesized as the search for more efficient antibacterial agents for use in PACT are developed.

Much more still needs to be done in the application of PACT in dermatological and control of infectious diseases, especially in the management of acne and skin infections in general. Given the wide range of bacteria, it has been noted that the potential application of PACT in the treatment of infectious diseases is still lagging even with the positive acceptance of photodynamic therapy in treatment for other diseases such as psoriasis and skin cancers. There is, therefore, a need for researchers to further explore this application.

While the application of the PACT systems has been extensively evaluated for topical/local approach for animal model evaluations, more studies on systemic application still need to be done to fully evaluate their *in vivo* stability and therapeutic modality. It is important to fully understand their mechanisms of action and fine-tune them appropriately to improve their sensitivity and selectivity. Notably, most studies discussed in this mini-review lacked toxicity data, and there is a need, therefore, for future studies to carry out toxicity studies (either short term or long term). This is very important because toxicity profile evaluation will go along way to bringing confidence in PACT systems before submitting the final products to regulatory endorsements.

Despite the gaps, the reported studies in this review indicate that there is a possibility of adding PACT systems to the current therapeutic arsenals for combating microbial resistance, especially where the conventional antimicrobials have failed. We believe that as the field of PACT continues to grow, the development of even more robust photosensitizers is more likely, based on the improved understanding of the specific action mechanisms and disease targeting ability of the developed systems. Such changes will lead to an increased role of PACT in the management of microbial infections.
